# Intradiscal electrothermal therapy in the treatment of chronic low back pain: Experience with 93 patients

**DOI:** 10.4103/2152-7806.67107

**Published:** 2010-08-04

**Authors:** Hsi-Kai Tsou, Shao-Ching Chao, Ting-Hsien Kao, Jia-Jean Yiin, Horng-Chaung Hsu, Chiung-Chyi Shen, Hsien-Te Chen

**Affiliations:** 1 Department of Neurosurgery, Taichung Veterans General Hospital, 160 Sec3 Chung-Kang Road, Taichung - 407 05, Taiwan, R.O.C; 2 Center for General Education, Jen-Teh Junior College of Medicine, Nursing and Management, 79-9 Sha-Luen Hu, Houloung Town, Miaoli County 356, Taiwan, R.O.C; 3 Department of Materials Science and Engineering, Feng Chia University, 100 Wen-Hwa Road, Taichung - 407 24, Taiwan, R.O.C; 4 Department of Orthopaedic Surgery, China Medical University Hospital, 2 Yue-Der Road, Taichung - 404 47, Taiwan, R.O.C

**Keywords:** Chronic low back pain, intradiscal electrothermal therapy, discogenic pain

## Abstract

**Background:**

Low back pain (LBP) has become a main cause of absenteeism and disability in industrialized societies. Chronic LBP is an important health issue in modern countries. Discogenic LBP is one of the causes of chronic low back pain. The management of chronic discogenic LBP has been limited to either conservative treatment or operative treatment. Intradiscal electrothermal therapy (IDET) is now being performed as an alternative treatment.

**Methods:**

Ninety-three consecutive patients undergoing IDET at 134 disc levels from October 2004 to January 2007 were prospectively evaluated. All patients had discogenic disease with chronic LBP, as determined by clinical features, physical examination and image studies, and had failed to improve with conservative treatment for at least 6 months. Follow-up period was from 1 week to 3 or more years postoperatively.

**Results:**

There were 50 male and 43 female patients, with a mean age of 46.07 years (range, 21-65 years). The results were classified as symptom free (100% improvement), better (≥50% improvement), slightly better (<50% improvement), unchanged and aggravated. Eighty-nine patients were followed up in the first week; of them, 77 (86.52%) patients had improvement (4, symptom free; 45, better; and 28, slightly better). The improvement rate gradually decreased to 80.90% in 1 year; and 73.91%, in 3 years.

**Conclusions:**

In conclusion, IDET offers a safe, minimally invasive therapy option for carefully selected patients with chronic discogenic LBP who have not responded to conservative treatment. Although IDET appears to provide intermediate-term relief of pain, further studies with long-term follow-up are necessary.

## INTRODUCTION

Chronic low back pain (CLBP) affects 60% to 85% of the population at least once in their life, and those whose problems become chronic account for 10% to 20%.[11,12] The use of heat energy to treat CLBP is an alternative to standard surgical procedures in certain patients. A common technique with this approach is termed intradiscal electrothermal annuloplasty or intradiscal electrothermal therapy (IDET). The rationale for heating intervertebral discs was strongly influenced by animal and clinical investigations testing the ability of heat to stabilize joints by modifying collagen. Since 1994, thermal capsulorrhaphy has been used alone or in combination with traditional surgical treatments to treat shoulder instabilities. Typically, the shoulder capsule is visualized through an arthroscope, and laser or radiofrequency heating devices are used to denature the collagen of the shoulder capsule, causing shrinkage.[4] Like the outer disc annulus, type I collagen in the shoulder capsule has a triple-helical configuration that is responsible for the molecule's ability to resist tensile forces. Heating is thought to first disrupt the weaker intramolecular bonds, unraveling and denaturing the triple helix. The stronger nonreducible intermolecular cross-links remain intact and thus the amount of collagen is unchanged. This “melting” into an amorphous state causes the observed “tissue shrinkage”and is a phase transition from a highly ordered crystalline structure to a random coil state.[4] This concept influenced the development of IDET. So IDET for discogenic low back pain was initially introduced by Saal *et al*. in 1997.[8]

In the past 3 years, we preferred the diagnostic criteria for lumbar discogenic pain according to the clinical features, physical examinations and image studies. The purpose of this study was to evaluate IDET's role as a definitive treatment for chronic discogenic low back pain in strictly selected patients. We hypothesized that IDET would be a beneficial additional treatment option for chronic discogenic low back pain. Here we present a single-arm, prospective analysis on 93 patients who had severe low back pain with or without radicular pain. They all were treated with application of IDET, and we evaluated the efficacy of IDET and the ability of patients to manage day-to-day tasks of life after IDET treatment.

## MATERIAL AND METHODS

### Patient selection

The IDET was performed as an outpatient procedure from October 2004 to January 2007 according to the inclusion and exclusion criteria listed in Table 1 and 2. We excluded patients with psychological distress / depressive mood or drug/ alcohol abuse. We don’t routinely evaluate psychological disorders by some questionnaire. There were 50 males and 43 females. Sixty-four patients were treated at the Department of Neurosurgery, Taichung Veterans General Hospital, Taiwan, and 29 patients were treated at the Department of Orthopedic Surgery, China Medical University Hospital, Taiwan. All patients had discogenic disease with CLBP, as determined by clinical features, physical examinations and image studies, and had failed to improve with conservative treatment administered for at least 6 months. The pain was provoked by lumbar hyperflexion, and they could not perform prolonged sitting or standing. All patients underwent dynamic X-ray and MRI study of the lumbar spine. They underwent IDET at one-to-three spinal levels according to their symptoms and the findings on imaging. The treatment levels of IDET are listed in Table 3. All patients' MRI of L-spine revealed at least one level of dehydration of intervertebral disc between L1-S1 and a suggestion of degenerated disc disease. Lumbar discography has been used extensively in the evaluation of low back pain since the early 1950s. The patient′s response to the injection is recorded as no pain, nonconcordant pain or concordant pain. The concordant pain is inferred to be present when the patient answers with a yes to these questions: Does each injection seem painful? If so, does the discomfort provoked by the injection seem similar to his/ her usual low back pain? Morphological characteristics of the disc being tested can be observed both fluoroscopically and with postdiscography CT scans. Despite its widespread use, discography remains controversial, primarily due to the lack of correlation between morphological findings and clinical symptoms and the reported high false-positive rates.[6] Nevertheless, we routinely get lumbar discography done by a radiologist to evaluate lumbar discogenic pain. Discography is performed by the injection of a non-irritating radiopaque dye, under X-ray guidance, into several discs of an awake subject. The central portion of the disc is percutaneously penetrated by a 22-gauge needle, usually from a posterolateral approach. The dye is then slowly injected into the disc. In skilled hands, needle placement with a local anesthetic at the skin puncture is quickly performed. The dye is then slowly injected into the nucleus of several lumbar discs in succession. The distribution of the dye in the disc is noted, as is the patient's response to injection. The patient is asked whether each injection seems painful and if so, whether the discomfort provoked by the injection seems similar (concordant) to his/ her usual low back pain. Therefore, the major criteria for a “positive” result of disc injection are pain of “significant” intensity on disc injections and a reported similarity of that pain to the patient′s usual clinical discomfort. The minor criteria for a “positive” result of disc injection are:

**Table 1:** Inclusion criteria

Candidate for IDET at one or two or three levels
Symptoms of degenerative lumbar disc disease of at least 6 months' duration
Failure to improve with a minimum of 6 months of conservative treatment (including pain medication and physical therapy)
Presenting with marked functional limitation
Sitting intolerance greater than standing intolerance
Presenting with predominant low back pain with or without referred leg pain
Negative straight leg raise and normal neurologic examination
The presence of degenerative disc disease on magnetic resonance scan with global disc degeneration or posterior or posterolateral annular tear evident
Minimum age, 18 years
Must be willing to comply with follow-up as per the protocol

**Table 2:** Exclusion criteria

Evidence of a large contained or sequestered herniation (small contained herniation was allowed)
Loss of more than 50% disc height at the target level
Severely disrupted disc (sufficient annular tissue required for safe catheter placement)
Neurogenic claudication due to spinal stenosis
Four or more symptomatic lumbar disc levels
Previous back surgery at any level of the lumbar spine
Spondylolisthesis at a symptomatic disc level
Psychological disorders that may impact treatment outcome

**Table 3:** Demographic and clinical features of the 93 patients

Feature	Number	Median [Range]
All patients	93	
Male	50	[23-65]
Female	43	[21-64]
Age (years, mean ± SD)		46.07 ± 13.13
Visual analog pain scale (0-100)		
Back	89	69.33 ± 16.08
Leg	75	64.00 ± 18.38
Discs level		
L_1_-_2_	1	
L_2_-_3_	3	
L_3_-_4_	20	
L_4_-_5_	65	
L_5_-S_1_	45	
Successive lumbar surgery	2	
Lost to follow-up	2

- negative control disc
- annular penetration of the dye
- single painful discs only
- positive pain behavioral signs during injection (Assessment of pain-related behavior was made on the basis of observations through a window from the start of the injection until its completion. Five types of pain-related behavior were recorded: guard/brace/withdraw, rubbing, grimacing, sighing, or verbalizing. The participant was considered to have demonstrated pain-related behavior if he or she exhibited two or more of these types of behavior.).[3]

The degree of pain was recorded by visual analog pain scale. Oswestry low back pain disability questionnaire has been designed to give information as to how back pain has affected ability to manage everyday tasks of life. So we used Oswestry low back pain disability questionnaire to evaluate the ability to manage everyday tasks of life.

### Methods

#### The IDET procedure

The IDET procedure uses a navigable intradiscal catheter with a thermal resistive coil. The procedure was performed under local anesthesia with lidocaine. Using fluorography, a 30-cm spineCATH catheter (Oratec Interventions, Inc., Menlo Park, CA) with a 5-cm active electrothermal tip was inserted anteriorly into the annulus or nucleus via a 17-gauge introducer. The active tip was advanced anterior-laterally inside the nuclear tissue and directed circuitously to return posteriorly, providing an ideal position to heat the entire posterior annulus. Once a satisfactory position was obtained in the anteroposterior, lateral views, the catheter was connected to a lead and passed to an independent technician. In all cases, the catheter tips were within 5 mm of the posterior vertebral margin upon review of saved fluoroscopic films. We used a standard protocol in which heating began at 65°C and was increased incrementally by 1°C every 30 seconds to achieve a final temperature of 90°C. The final temperature was maintained for 4 minutes, giving a total treatment time of 16.5 minutes.

## RESULTS

The demographic and clinical features of the patients are listed in Table 3. The patients underwent IDET at one-to-three spinal levels unilaterally according to the image findings and the most painful side. There were 50 male and 43 female patients, with a mean age of 46.07 ° 13.13 years (range, 21-65 years). The treatment disc levels were 134 levels, and the level of most of the patients was L4-L5. The mean preoperative visual analog pain scale score of patients who had back and leg pain was 69.33 ° 16.08 and 64.00 ° 18.38 (0-100), respectively. Follow-up period ranged from 1 week to 3 years postoperatively.

**Figure 1:**
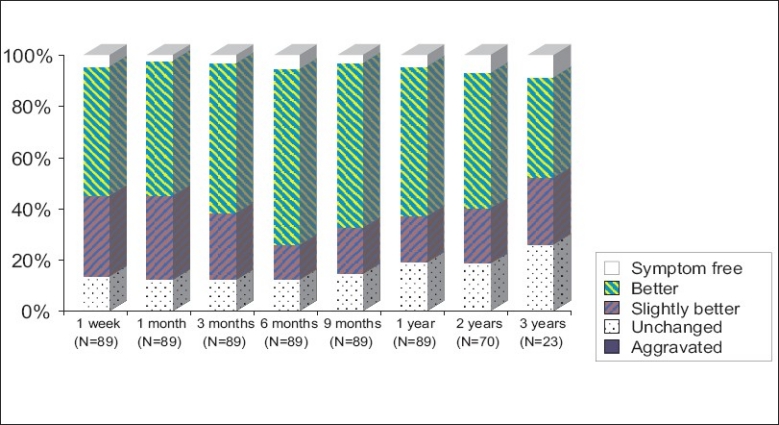
Low back results after IDET

**Figure 2:**
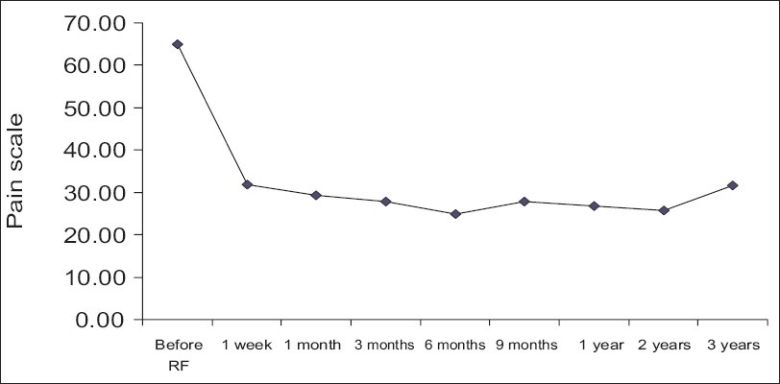
Low back pain scale distribution with improvement ≥50% and lasting for more than 3 months

**Figure 3:**
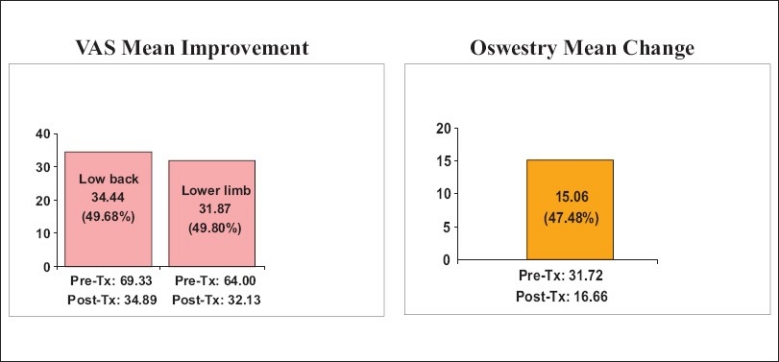
VAS mean improvement in low back and lower limbs pain and Oswestry mean change from pre-IDET to 1 year post-IDET

The results were classified as symptom free (100% improvement), better (≥50% improvement), slightly better (<50% improvement), unchanged and aggravated. The results of low back pain after IDET are listed in Table 4. After IDET, of the 89 patients, 49 (55.06%) had initial improvement of ≥50% in the first-week follow-up, whereas 66 (74.16%) patients had initial improvement of ≥50% at the 6-month follow-up. After 2-years follow-up, 42 (60.00%) of the 70 patients after IDET were relieved of pain ≥50%. The results with regard to relief in low back pain are diagrammatically shown in Figure 1. To analyze all patients with low back pain relief ≥ 50% for more than 3 months, the most effective period was 6 months post-operation [Figure 2]. A mean improvement of 49.68% in the visual analog scale (VAS) was obtained between pre-and post-IDET treatment in the 1-year outcome [Figure 3]. No complication was found among these patients.

**Table 4:** Low back pain results after IDET

	1 week (n=89/89)	1 month (n=89/89)	3 months (n=89/89)	6 months (n=89/89)	9 months (n=89/89)	1 year (n=89/89)	2 years (n=70/89)	3 years (n=23/89)
Worse	0	0	0	0	0	0	0	0
0%	12	11	11	11	13	17	13	6
0%< and<50%	28	29	23	12	16	16	15	6
50%≤ and<100%	45	47	52	61	57	52	37	9
100%	4	2	3	5	3	4	5	2
Improvement rate (%)*	86.52	87.64	87.64	87.64	85.39	80.90	81.43	73.91
Satisfactory rate (%)*	55.06	55.06	61.80	74.16	67.42	62.92	60.00	47.83

* Improvement was defined as patient having pain relief of >0%. Satisfaction was defined as patient having pain relief of ≥50%.

**Figure 4:**
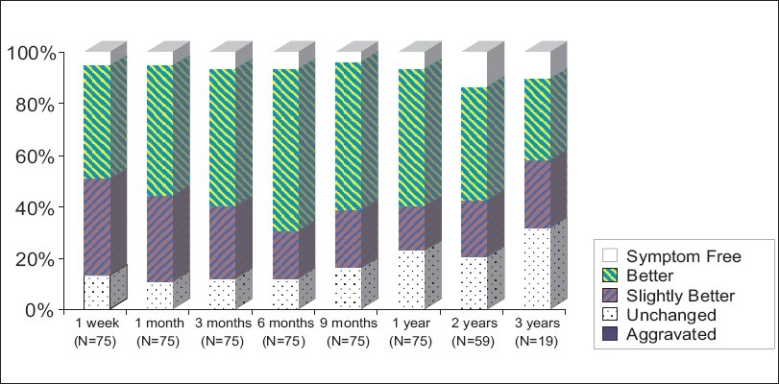
Lower limb results after IDET

**Figure 5:**
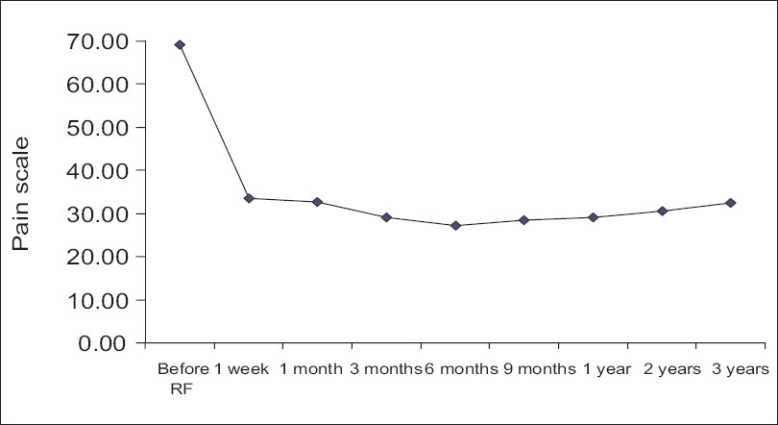
To analyze all patients with lower limbs pain relief ≥ 50% for more than three months, the most effective period was all located in post-operation six months later

The results with regard to post-IDET lower limb pain are listed in Table 5. After IDET, of the 75 patients, 37 (49.33%) had initial improvement of ≥50% in the first-week follow up, whereas 52 (69.33%) patients had initial improvement of ≥50% at the 6-month follow-up. After IDET, at the 2-year follow-up, 34 (57.63 %) of the 59 patients had pain relief of ≥50%. The results with regard to relief in lower limbs pain are diagrammatically shown in Figure 4. To analyze all patients with lower limbs pain relief ≥ 50% for more than three months, the most effective period was all located in post-operation six months later [Figure 5]. There was 49.80% improvement in mean VAS levels from pre-IDET to 1 year post-IDET [Figure 3]. No complication was found among these patients.

**Table 5:** Lower limb pain results after IDET

	1 week (n=75/75)	1 month (n=75/75)	3 months (n=75/75)	6 months (n=75/75)	9 months (n=75/75)	1 year (n=75/75)	2 years (n=59/75)	3 years (n=19/75)
Worse	0	0	0	0	0	0	0	0
0%	10	8	9	9	12	17	12	6
0%< and<50%	28	25	21	14	17	13	13	5
50%≤ and<100%	33	38	40	47	43	40	26	6
100%	4	4	5	5	3	5	8	2
Improvement rate (%)*	86.67	89.33	88.00	88.00	84.00	77.33	79.66	68.42
Satisfactory rate (%)*	49.33	56.00	60.00	69.33	61.33	62.92	60.00	42.11

* Improvement was defined as patient having pain relief of >0%. Satisfaction was defined as patient having pain relief of ≥50%.

A significant improvement in physical functioning was demonstrated by the IDET-treated group as measured by the physical functioning scale of the Oswestry low back pain disability questionnaire. A significant improvement was detected at 6 months post-treatment. The average of Oswestry scale scores of the 89 patients pre-IDET was 31.72, which decreased to 16.66 after 1 year of IDET treatment. There was 47.48% improvement from pre-IDET to 1 year post-IDET [Figure 3].

The data were analyzed by using paired t tests to evaluate the therapeutic effects of IDET treatments. We constructed a 99% (α= 0.01) confidence interval, and we found it would reject null hypotheses H_0_. Thus, IDET treatments showed significant beneficial effects.

## DISCUSSION

Intradiscal electrothermal heating treatment is a minimally invasive procedure used to treat patients with low back and referred leg pain, but how or why heating decreases discogenic pain is unclear.[6,10] There has been much written about the proposed mechanism of action of IDET. Proposed mechanisms include alteration of spinal segment mechanics via collagen modification, coagulation of annular nociceptors leading to contraction of collagen,[9] biochemical mediation of inflammation,[9,10] stimulation of an outer annular healing response,[9,10] induced healing of annular fissures,[9,10] decreased intradiscal pressure [7] and cauterization of vascular ingrowth.[9,10] None of these proposed mechanisms has been proven. In addition, the original concept that annular heating would cause beneficial collagen modification remains unproven, and animal studies suggest that instead there may be a decrease in motion stability.[6] It is clear that further studies in basic science are required to explain the mechanism of action of IDET.

Derby *et al*. recently published a review paper about evidence-informed management of chronic low back pain with intradiscal electrothermal therapy.[4] They made analysis of systematic reviews and randomized controlled trials about the efficacy of IDET for CLBP patients. Appleby *et al*. recently published a meta-analysis [2] of 17 IDET studies with follow-up of 6 to 24 months and validated outcome measures for pain or function. The pooled analysis from these studies found a mean decrease in visual analog scale of 2.9, a mean decrease in short-form 36 (SF-36) physical function of 21.1, a mean decrease in SF-36 bodily pain of 18, and a mean decrease in Oswestry Disability Index score of 7.0 — all of which were statistically significant. A review by Anderson *et al*. compared validated outcome measurements of 18 IDET studies with the same outcome measurements reported by 33 studies of fusion for degenerative disc disease.[1] The overall median decrease in pain reported after IDET was very similar to that after spinal fusion. In contrast, a prospective, randomized, double-blind, placebo-controlled trial of IDET for the treatment of CDLBP was designed by Freeman BJ. Fifty-seven patients were randomized with a 2:1 ratio: 38 to IDET and 19 to sham procedure (placebo). An independent technician connected the catheter to the generator and then either delivered electrothermal energy (active group) or did not (sham group). Surgeon, patient, and independent outcome assessor were all blinded to the treatment. This study demonstrates no significant benefit from IDET over placebo.[5] A meta-analysis by Freeman reviewed the same studies carried out by Appleby *et al*. but reached different conclusions.[5] Their review reported a mean improvement in VAS scores of 3.4, and only slightly lower mean Oswestry Disability Index (ODI) improvement of 5.2. In addition, only 13% to 23% of the patients treated with IDET required surgery. Despite these seemingly positive findings in studies of basic science required to explain the mechanism of action of IDET, Freeman concluded that “the evidence for efficacy of IDET remains weak and has not passed the standard of scientific proof."

In our paper, we have described treatment of 93 cases with 134 degenerated disc levels by applications of IDET procedure and tried to analyze the results. The single-arm, prospective analysis showed that the application of IDET is a safe and useful intervention for chronic low back pain. The satisfactory pain relief obtained in the majority (more than 50%) of our patients only with chronic low back pain and lower limb pain justifies a study of at least 2 years. Approximately more than half of the patients were satisfied with their outcomes, and nearly all continued to follow up approximately 2 years post-IDET in this study cohort. These results are a great contrast to those of the previous studies evaluating outcomes of the IDET procedure.

## CONCLUSION

Although the initial research appeared promising, IDET should be scrutinized more closely using controlled studies. On more long-term follow-up of this patient population, we find that this procedure may be less effective than previously reported. Only through the use of a blinded, treatment-uncontrolled study with long-term follow-up can questions regarding the efficacy of IDET be fully answered.
